# Protective Effects of Thyroid Hormone Deprivation on Progression of Maladaptive Cardiac Hypertrophy and Heart Failure

**DOI:** 10.3389/fcvm.2021.683522

**Published:** 2021-07-30

**Authors:** Helena Kerp, Georg Sebastian Hönes, Elen Tolstik, Judith Hönes-Wendland, Janina Gassen, Lars Christian Moeller, Kristina Lorenz, Dagmar Führer

**Affiliations:** ^1^Department of Endocrinology, Diabetes and Metabolism, University Hospital Essen, University of Duisburg-Essen, Essen, Germany; ^2^Leibniz-Institut für Analytische Wissenschaften-ISAS-e.V., Dortmund, Germany; ^3^Institute of Pharmacology and Toxicology, University of Würzburg, Würzburg, Germany

**Keywords:** thyroid hormones, maladaptive cardiac hypertrophy, pressure-overload, heart failure, mice

## Abstract

**Purpose:** Thyroid hormones (TH) play a central role for cardiac function. TH influence heart rate and cardiac contractility, and altered thyroid function is associated with increased cardiovascular morbidity and mortality. The precise role of TH in onset and progression of heart failure still requires clarification.

**Methods:** Chronic left ventricular pressure overload was induced in mouse hearts by transverse aortic constriction (TAC). One week after TAC, alteration of TH status was induced and the impact on cardiac disease progression was studied longitudinally over 4 weeks in mice with hypo- or hyperthyroidism and was compared to euthyroid TAC controls. Serial assessment was performed for heart function (2D M-mode echocardiography), heart morphology (weight, fibrosis, and cardiomyocyte cross-sectional area), and molecular changes in heart tissues (TH target gene expression, apoptosis, and mTOR activation) at 2 and 4 weeks.

**Results:** In diseased heart, subsequent TH restriction stopped progression of maladaptive cardiac hypertrophy and improved cardiac function. In contrast and compared to euthyroid TAC controls, increased TH availability after TAC propelled maladaptive cardiac growth and development of heart failure. This was accompanied by a rise in cardiomyocyte apoptosis and mTOR pathway activation.

**Conclusion:** This study shows, for the first time, a protective effect of TH deprivation against progression of pathological cardiac hypertrophy and development of congestive heart failure in mice with left ventricular pressure overload. Whether this also applies to the human situation needs to be determined in clinical studies and would infer a critical re-thinking of management of TH status in patients with hypertensive heart disease.

## Introduction

Despite great therapeutic advances, cardiovascular diseases remain the most common cause of death, globally (1). Many clinical and animal studies have described a close association of thyroid dysfunction with cardiovascular diseases (2–5). Both systemic hyperthyroidism and hypothyroidism have been identified as risk factors for heart failure. However, the precise role of TH in development and progression of heart failure is still not fully understood.

TH exert positive inotropic and chronotropic effects in the heart *via* direct and indirect mechanisms (3, 6–8). For example, TH stimulate cardiac protein synthesis, promote physiological cardiac growth and angiogenesis, and reduce cardiac afterload by decreasing arterial vascular smooth muscle tone (2, 9). In the heart, these physiological TH effects involve sarcomeric proteins, such as myosin heavy chain alpha (*MYH6*) and beta (*MYH7*), regulators of calcium homeostasis such as sarcoplasmic/endoplasmic reticulum Ca^2+^ ATPase 2a (*SERCA2a*) and phospholamban (*PLN*) as well as beta1-adrenoreceptors (10, 11). For a limited duration, treatment with TH has been shown to improve cardiac output, reduce afterload, and lead to a compensated, so-called physiological or “athletic,” cardiac hypertrophy (3, 12–18). These effects could even be shown in relation to cardiac diseases in a small patient cohort with advanced heart failure (19) and in patients with dilated cardiomyopathy (20). While this supports a beneficial role of TH in heart failure, large cohort studies and comprehensive experimental characterization of heart disease under low and high TH availability are lacking. Thus, the aim of our study was to investigate longitudinally whether modulation of TH status at early stages in an already diseased heart could affect cardiac outcome. To this end, we induced chronic left ventricular pressure overload in mice by transverse aortic constriction (TAC) and studied changes in heart function and structure under TH deprivation and high TH availability over a period of 4 weeks. Modulation of thyroid status commenced 1 week after TAC, when first signs of cardiac hypertrophy and impaired cardiac function have evolved (21). In this heart disease model, we show that reducing TH availability effectively slows down pathological cardiac growth and improves cardiac function even under continued left ventricular pressure overload, while opposite effects occur with increased TH availability.

## Materials and Methods

### Animals

Male wild-type C57BL/6JRj mice (Janvier Labs, France) aged 2 months (*n* = 6–9 animals per treatment) were studied. All mice were housed in groups of three to five animals in temperature (23 ± 1°C)- and light (inverse 12:12-h light–dark cycle)-controlled conditions. Food and water were provided *ad libitum*. All animal experiments were performed in accordance with the German regulations for Laboratory Animal Science (GVSOLAS) and the European Health Law of the Federation of Laboratory Animal Science Associations (FELASA). The protocols for animal studies were approved by the Landesamt für Natur, Umwelt und Verbraucherschutz Nordrhein-Westfalen, Germany (LANUV-NRW, AZ 84-02.04.2016.A261).

### Transverse Aortic Constriction

Mice were subjected to TAC to induce chronic left ventricular pressure overload using a 27-gauge needle for 4 weeks as described previously (22). Before TAC, as well as 2 and 4 weeks after TAC, echocardiography was performed. Hearts were isolated for biochemical, histological, and weight analyses. The surgeon was blinded to treatment groups. Banded mice with an aortic pressure gradient below 60 mmHg were excluded from this study.

### Echocardiography

Transthoracic echocardiograms of male mice were performed in a blinded manner using the Vevo2100 high-resolution imaging systems (VisualSonics) and a 30-MHz probe. Values for end-diastolic and -systolic intraventricular septal (IVSd/IVSs) and left ventricular posterior wall thicknesses (LVPWd/LVPWs) as well as end-diastolic and -systolic left ventricular internal diameters (LVIDd/LVIDs) were obtained from 2D M-mode images in the short-axis view at the proximal level of the papillary muscles. Peak blood flow velocities at the site of TAC [*V*_max_ (mm/s)] were derived from pulsed-wave Doppler measurements. Fractional shortening (FS) as a measure for cardiac contractility (23), ejection fraction (EF), and aortic pressure gradients (mmHg) were calculated by VisualSonics Cardiac Measurements software. The data shown represent averages of at least six cardiac cycles per animal. The investigator was blinded to experimental settings during measurements and data analysis. Mice with heart rates below 450 bpm were excluded from the study.

### Treatment

**Figure 1:**
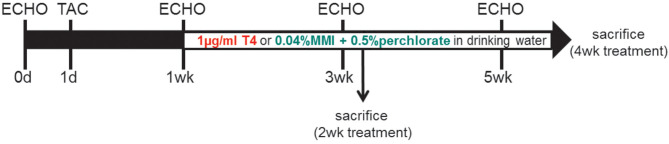
Study design: Two-month-old male C57Bl/6 mice were subjected to TAC at experimental day 1. One week after banding, when cardiac hypertrophy had developed, animals were exposed to either TH treatment or TH deprivation by 1 μg/ml T4 or LoI/MMI/${\text{ClO}}_{4}^{-}$ in drinking water, respectively. Control mice were maintained on normal chow and drinking water without supplements. Serial echocardiography (ECHO) was conducted at day 0 and at 1, 3, and 5 weeks (wk) after TAC surgery. Mice were sacrificed at 2 and 4 weeks after start of treatment protocol (3 and 5 weeks after TAC).

Mice were subjected to three treatment protocols: (1) For increased TH availability (TH high) mice were fed normal diet (MD.1572, Envigo, USA) and received 1 μg/ml T4 (Sigma-Aldrich (T2376), USA; stock solution: 100 μg/ml T4 solved in 40 mM NaOH and 2 g/L bovine serum albumin) in drinking water. (2) For TH deprivation (TH low), animals were fed a low-iodine diet (LoI; MD.1571, Envigo, USA) and received drinking water supplemented with anti-thyroid drugs 0.04% methimazole [MMI, Sigma-Aldrich (301507), USA], 0.5% sodium perchlorate [${\text{ClO}}_{4}^{-}$, Sigma-Aldrich (310514), USA]), and 0.3% saccharine as sweetener [Sigma-Aldrich (240931), USA] (LoI/MMI/${\text{ClO}}_{4}^{-}$). (3) In the control group (ctrl), animals were fed a normal diet (MD.1572, Envigo, USA) and received drinking water without supplements. Other than the low iodine content in the TH deprivation group, caloric and nutritional composition of chow was comparable in all three mouse groups. The treatment protocols started 1 week after TAC surgery and were continued for 2 or 4 weeks as indicated (Figure 1).

Male mice under chronic high or low TH availability without TAC surgery have been extensively studied by us earlier (24–26) and TAC sham operation has previously been shown not to affect parameters of cardiac function or morphology (22, 27, 28). For these reasons and in line with the “3R concept” in animal research, the present study was done without including an additional sham-operated control group.

### Organ Isolation and Serum Measurements

Mice were deeply anesthetized at 3 or 5 weeks after TAC surgery, i.e., 2 or 4 weeks after start of treatment protocols, by ip injection of 200 μl of Ketamine/Xylazine mixture [150 μl of 100 mg/ml Ketamine (Bela-pharm, Germany) and 50 μl of 20 mg/ml Xylazine (Ceva, Germany)], and final blood was obtained by right ventricular heart puncture. For tissue collection, mice were perfused with heparinized saline through a needle placed in the left heart ventricle. Hearts were shock frozen in liquid nitrogen, and stored at −80°C until further processing or fixed in 4% buffered formalin (Formafix, Germany). Final blood samples were stored 30 min on ice and centrifuged, and free triiodothyronine (fT3), free thyroxine (fT4), and total T4 (TT4) concentrations in serum of mice were measured using commercial ELISA kits according to the manufacturer's instructions (DRG Instruments GmbH, Marburg, Germany). Detection limits were 0.5 μg/dl, 0.05 ng/dl, and 0.05 pg/ml for TT4, fT4, and fT3, respectively. Values below were calculated from the standard curve.

### RNA Isolation and qRT-PCR

Total RNA from hearts was isolated and reverse transcribed into cDNA as previously described (29). In compliance with the MIQE guidelines for RT-PCR, we used a set of three reference genes to assure accurate normalization and calculation, *Gapdh* (glyceraldehyde-3-phosphate dehydrogenase), *Rn18s* (18S ribosomal RNA), and *Polr2a* (polymerase RNA II). Primer sequences of all quantified genes are listed in Supplementary Table 1. Analysis and calculation of the fold change in gene expression were done on *Ct* ≤ 35 using the efficiency corrected method.

### TUNEL Assay

After dewaxing and rehydration, tissue sections were permeabilized with proteinase K. For TUNEL staining, an *in situ* cell death detection kit (Sigma-Aldrich, USA) was used according to the manufacturer's protocol. Pretreatment with DNase I served as a positive control and TUNEL reaction mixture lacking terminal transferase (TdT) served as a negative control. Cell nuclei and membranes were counterstained with DAPI (D1306, Thermo Fisher Scientific, USA) and wheat germ agglutinin (W11261, Thermo Fisher Scientific, USA). For quantification, blind analysis of sections pictured with the Olympus BX51 upright microscope (Olympus, Germany) was conducted.

### Histological Staining and Quantification of Cardiomyocyte Sizes

Formalin-fixed hearts were embedded in paraffin and 5-μm-thick sections were used for staining. Hematoxylin and eosin (H&E) staining was performed for determination of cardiomyocyte sizes and Sirius Red for fibrosis as described previously (27). For quantification, left ventricular sections were recorded using Olympus BX51. Cross-sectional areas of cardiomyocytes in H&E- or wheat germ agglutinin-stained vertical sections were determined (*n* = 37–107 cells per animal) using ImageJ. Only cells with visible central nucleus were included. Fibrosis was calculated as the ratio of red stained/myocardial area *via* Adobe Photoshop. Analysis and quantification were done in a blinded manner.

### Immunoblot

Extraction of whole protein lysates, protein quantification, and immunoblotting were performed as described previously (30). The following antibodies were used: anti-AKT (9,272, Cell Signaling, 1:1,000, rabbit), anti-p-AKT (S473) (9271S, Cell Signaling, 1:1,000, rabbit), anti-ERK 1/2 (4695S, Cell Signaling, 1:1,000, rabbit), anti-p-ERK 1/2 (T202/Y204) (4370T, Cell Signaling, rabbit), anti-mTOR (2972S, Cell Signaling, 1:1,000, rabbit), anti-p-mTOR (Ser2448) (5536S, Cell Signaling, 1:1,000, rabbit), anti-BAX (2,772, Cell Signaling, 1:1,000, rabbit), anti-GAPDH (ACR001P, Acris Antibodies, 1:6,000, mouse), anti-mouse IgG (7,076, Cell Signaling, 1:2,000, goat), and anti-rabbit IgG (7,074, Cell Signaling, 1:2,000, goat). Visualization was done by luminescence using the Immun-Star™ WesternC™ Kit (BioRad, USA) and VersaDoc System (BioRad, USA). Differences in protein expression levels were quantified by densitometry using the Image Lab™ Software (BioRad, USA).

### Two-Photon and Polarized Light Microscopy

Cardiac fibrotic tissue was visualized by two-photon microscopy using Leica TCS SP8 microscope and Leica Application Suite (LAS X) software, equipped with a Chameleon Vision II Titan-Sapphire laser and a 25 × HCX IRAPO L water-immersion objective with a NA of 0.95. Autofluorescence of the heart and second harmonic signal (SHG) were detected using an excitation wavelength of 900 nm. A hybrid detector (HyD), bandpass emission filter 525/50 was used for autofluorescence detection, and SHG was detected using a hybrid detector (HyD), bandpass emission filter 460/50. Z-stacks of 12 μm with a spacing of 1 μm were acquired and pictures were projected with ImageJ. Cardiac fibrotic tissues from all six groups were additionally examined with a polarization microscope to assess the birefringence pattern of collagen fibers. Polarization imaging was performed using a Leica TCS SP8 DMi8 CS Bino DLS microscope with a magnification of 20 ×. Single random *xy* mappings were acquired in 512 × 512 image format, where *x* and *y* described the length and width of the mapping in pixels. Each image was acquired in polarized and bright-field light. Images were captured and exported using the Leica Application Suite (LAS X) integrated software.

### Statistical Analysis

Statistical analysis was performed using GraphPad Prism 6 Software. Two-way ANOVA followed by Tukey's *post-hoc* analysis or unpaired Student's *t*-test was applied as indicated. Values of ^*^p < 0.05, ^**^p < 0.01, ^***^p < 0.001, and ^#^*p* < 0.0001 were considered statistically significant. Outliers were identified using GraphPad Outlier test and were excluded if significantly (*p* < 0.05) different from the group.

## Results

To investigate how TH status impacts progression of pathological cardiac hypertrophy and development of heart failure, we used a heart disease mouse model with chronic left ventricular pressure overload induced by ligation of the transverse aorta. Well-characterized features of this model are pathological growth of the heart, i.e., maladaptive cardiac hypertrophy associated with interstitial fibrosis and apoptosis that subsequently leads to decreased FS and congestive heart failure (31). In this model and at an early stage of evolving cardiac disease at 1 week after TAC surgery, we changed TH status in mice by either oral T4 supplementation or TH deprivation through anti-thyroidal drug treatment (LoI/MMI/${\text{ClO}}_{4}^{-}$) of mice. As depicted in the study design (Figure 1), these treatments were conducted over 4 weeks.

**Figure 2:**
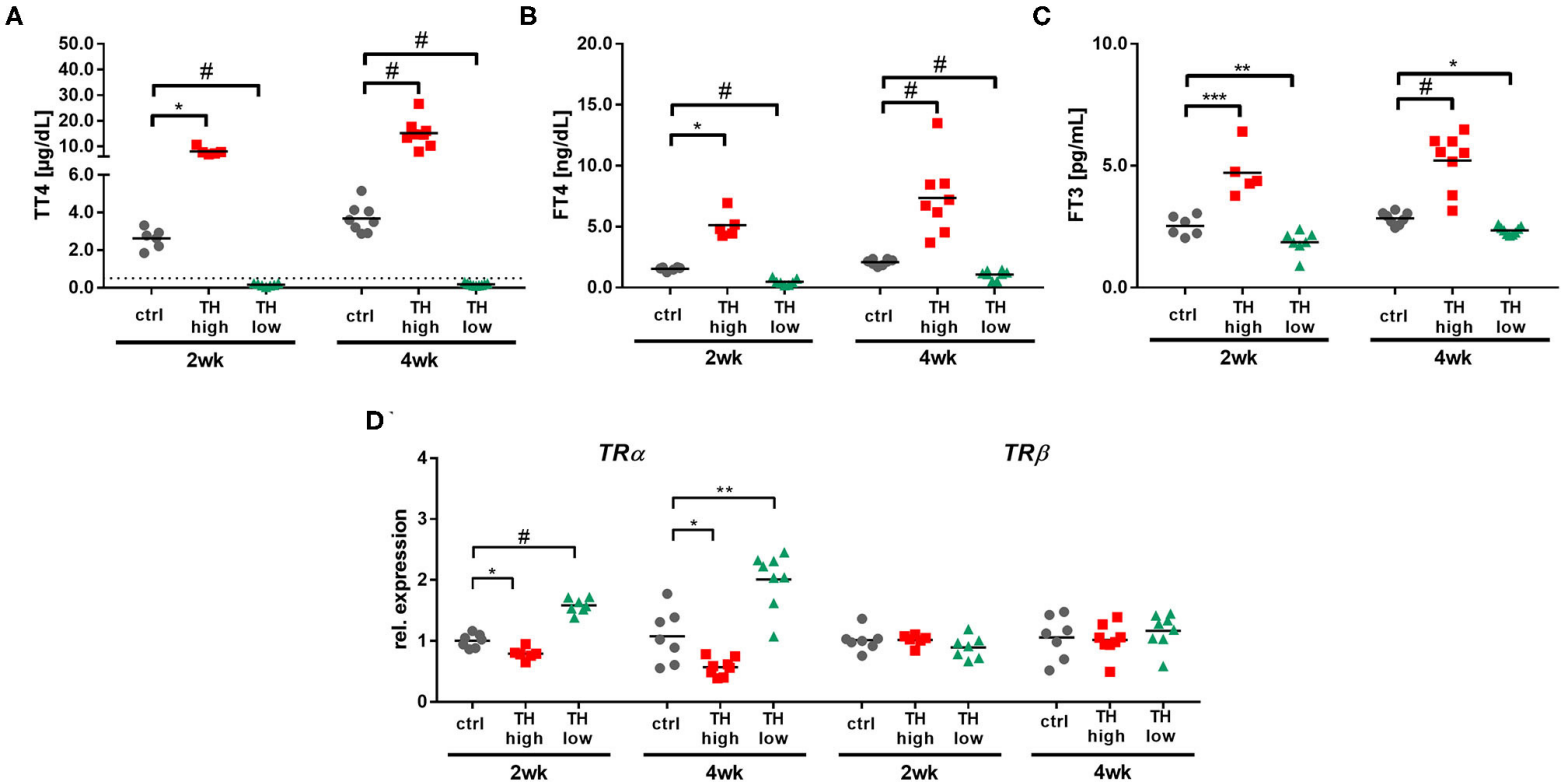
Confirmation of increased (TH high) or decreased (TH low) TH serum concentrations in T4- or LoI/MMI/${\text{ClO}}_{4}^{-}$-treated TAC mice (*n* = 5–9 per group) compared to euthyroid control TAC mice (ctrl). Dotted line represents detection limit of assay and values below were calculated from the standard curve. Serum TH concentrations (TT4, fT4, and fT3; **A–C**) and cardiac TRα and TRβ expression **(D)** were measured after 2 weeks and 4 weeks of treatment. Scatter dot plot and mean in all panels, *p < 0.05, **p < 0.01, ***p < 0.001, ^#^*p* < 0.0001 by two-way ANOVA and Tukey's *post-hoc* analysis for TH serum measurement and by unpaired *t*-test for qRT-PCR, ctrl, control; wk, weeks.

Notably, TAC surgery *per se* had no impact on serum TH status in mice, since TT4, fT4, and fT3 serum concentrations in TAC operated control mice were comparable to TH levels in 2-month-old TAC naïve mice (24). In contrast, T4 treatment or TH deprivation led to the expected alterations in TH serum concentrations with a hyperthyroid and hypothyroid serum status in TAC mice at 2 and 4 weeks [Figure 2; (32, 33)]. Additionally, cardiac TH receptor expression α (TRα) and β (TRβ) assessed by qRT-PCR showed decreased *TR*α availability upon hyperthyroidism and increased during TH deprivation. *TR*β expression was not altered by TH status (Figure 2D).

### TH Deprivation Stops Progression of Maladaptive Cardiac Hypertrophy and Prevents Development of Heart Failure

**Figure 3:**
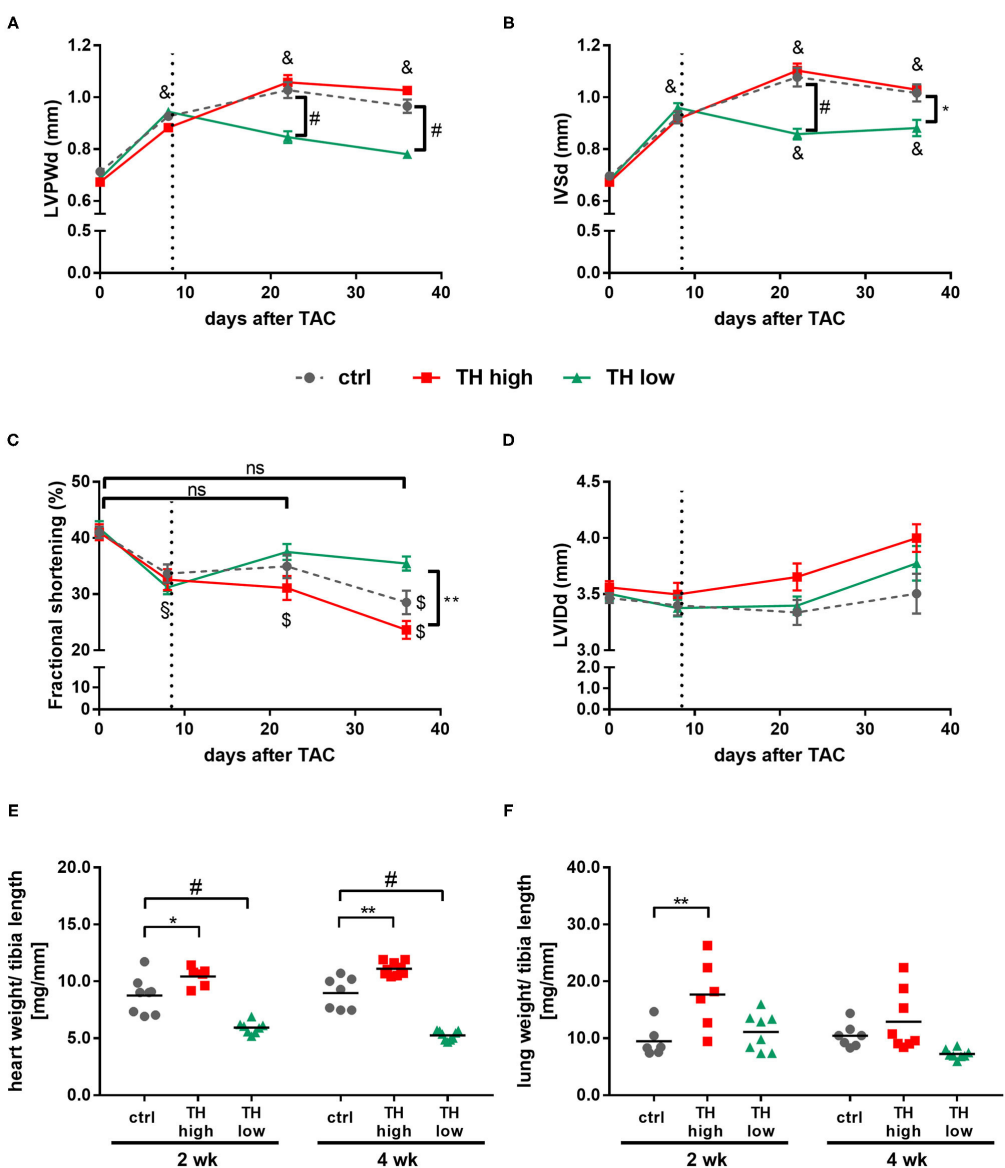
TH deprivation stops progression of maladaptive cardiac hypertrophy, improves fractional shortening, and effectively prevents development of heart failure. Echocardiography was conducted prior to TAC and 1, 3, and 5 weeks after surgery. Diastolic left ventricular posterior wall (LVPWd; **A**), intraventricular septum (IVSd; **B**) thickness, fractional shortening **(C)**, and diastolic left ventricular inner diameter (LVIDd; **D**) were determined in all mice. Values are indicated as mean ± SEM, for *n*, see Supplementary Table 2. *p < 0.05 TH high vs. TH low, **p < 0.01 TH high vs. TH low, ^#^*p* < 0.0001 to TH low at indicated time, ^§^*p* < 0.05 to baseline, ^$^*p* < 0.001 to baseline, ^&^*p* < 0.0001 to baseline by two-way ANOVA and Tukey's *post-hoc* analysis, ctrl, control; ns, not significant; wk, weeks. Dotted line represents start of T4 or LoI/MMI/${\text{ClO}}_{4}^{-}$ treatment. Analysis of heart weight-to-tibia length ratio (**E**, *n* = 6–9) and lung weight-to-tibia length ratio (**F**, *n* = 6–8) was determined at 2 and 4 weeks after start of treatment protocol. Scatter dot plot and mean, *p < 0.05, **p < 0.01, ^#^*p* < 0.0001 by two-way ANOVA and Tukey's *post-hoc* analysis.

The progression of pathological cardiac hypertrophy and cardiac dysfunction in response to TAC was monitored by serial echocardiograpy. One week after surgery—before manipulation of TH status was started—wall thickness of left ventricles was already significantly increased in TAC mice as depicted by the diameter of posterior and septum walls compared to corresponding baseline values prior to surgery (Figures 3A,B). Additionally, all TAC-treated mice showed significantly reduced FS (Figure 3C); however, no dilatation of the left ventricle was yet evident (Figure 3D).

In the following 4 weeks, the increase in wall thickness continued in control mice and was accompanied by a further significant decrease in FS over time. Interestingly, no differences in FS were observed between control and T4-treated mice (Figures 3A–C). In contrast, TH deprivation stopped progression of pathological cardiac hypertrophy and improved cardiac performance with a marked increase in FS after 2 and 4 weeks of treatment compared to control and T4-treated mice [Figure 3C and Supplementary Figure 1]. These differences in the progression of TAC-induced cardiac hypertrophy and changes in cardiac function under increased or decreased TH availability were also illustrated by changes in heart weight. Thus, after 2 and 4 weeks, heart weight-to-tibia length ratios in TH-deprived mice were significantly lower, while those for T4-treated mice were significantly higher compared to control animals (Figure 3E). Cardiomyocyte cross-sectional areas correlated with heart weight and were significantly smaller in TH-deprived mice compared to controls after 4 weeks of treatment [Supplementary Figure 2]. Furthermore, significantly increased lung weight, a functional indicator of congestive heart failure, was noted in T4-treated TAC mice, an effect that was significant after 2 weeks of treatment compared to TAC controls (Figure 3F). In line with these findings—even though not of statistic relevance−2 of 17 T4-treated mice died, while all TH-deprived (*n* = 17) and all control (*n* = 16) mice survived the 4-week treatment period after TAC.

### T4 Treatment in Chronic Pressure Overload Increases Cardiomyocyte Death Rate and Expression of Heart Failure Markers

Interstitial fibrosis due to proliferation of fibroblasts, collagen accumulation, and cardiomyocyte death causes cardiac muscle stiffness (34). To evaluate whether tissue remodeling contributes to the functional outcome, mouse hearts were histologically analyzed for interstitial fibrosis, apoptotic cell death by terminal deoxynucleotidyl transferase dUTP nick-end labeling (TUNEL), and pro-apoptotic signaling by protein expression of pro-apoptotic Bcl2-associated protein X (BAX).

**Figure 4:**
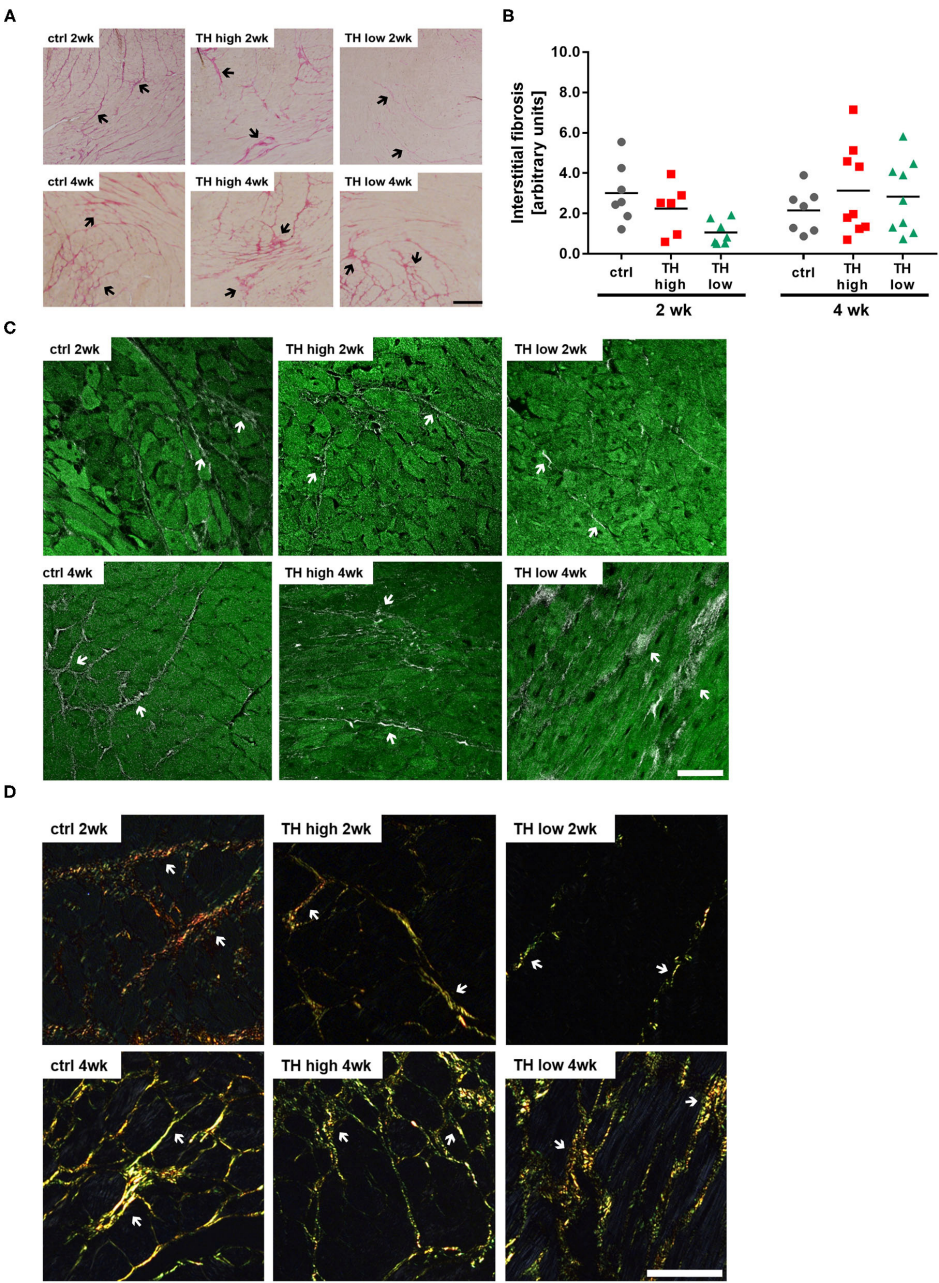
T4 status has no effect on fibrosis in TAC-operated hearts. Representative pictures of Sirius Red-stained sections (**A**, scale bar = 200 μm, black arrows indicate fibrotic fibers) and relative quantification of interstitial fibrosis (**B**, *n* = 6–9). Overlaid second harmonic generation images (gray) and autofluorescence signals (green) (**C**, scale bar = 50 μm, white arrows indicate fibrotic fibers) of heart slices were compared to polarized light imaging (**D**, scale bar = 50 μm, white arrows indicate fibrotic fibers).

**Figure 5:**
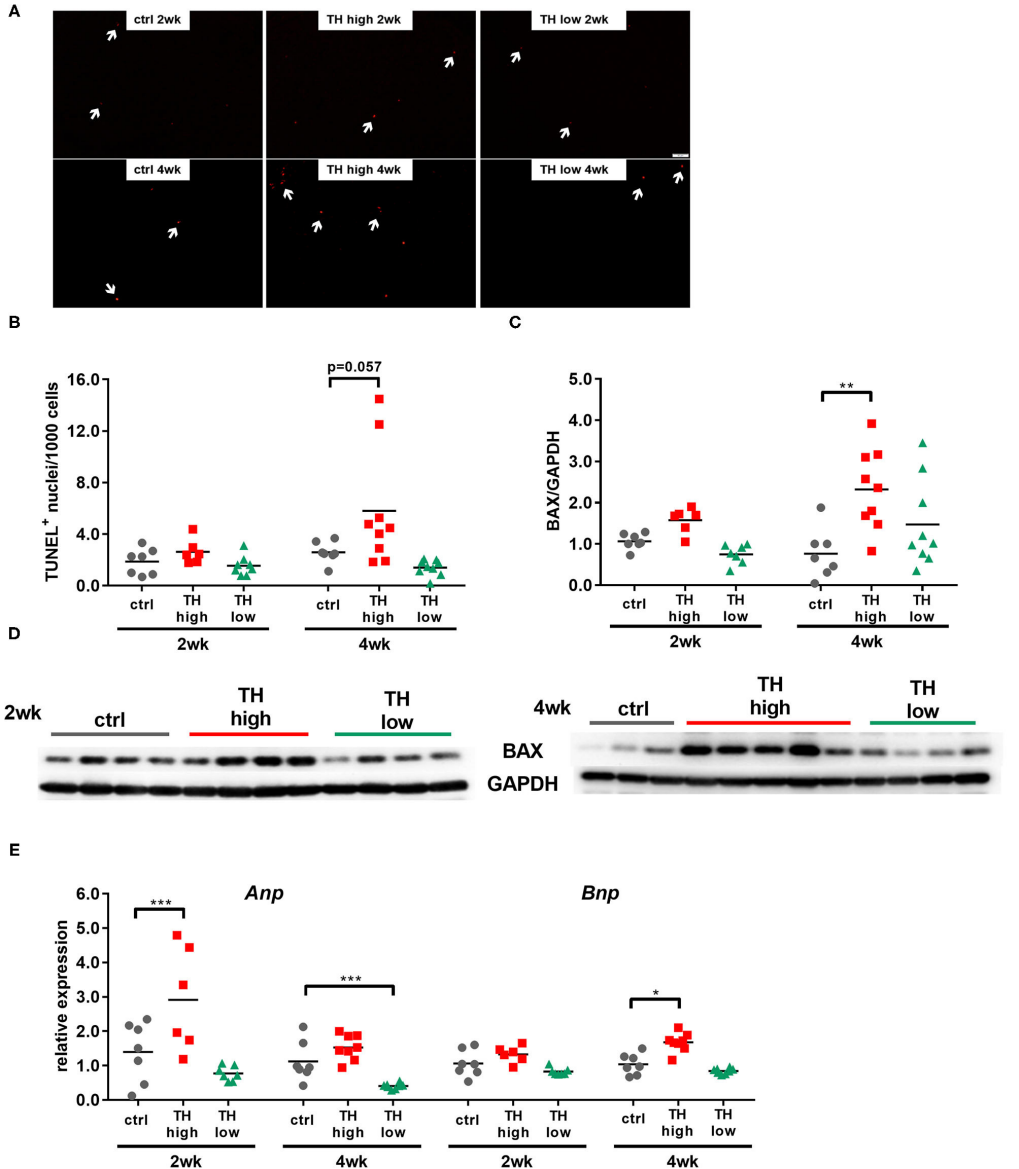
Cardiomyocyte apoptosis and expression of heart failure markers are increased in T4-treated TAC-operated hearts. TUNEL-positive nuclei (**A**, scale bar = 50 μm, white arrows indicate TUNEL-positive nuclei) were quantified in heart sections (**B**, *n* = 6–9), and cardiac protein expression of Bax was assessed by Western blotting after 2 and 4 weeks of treatment (**C**, **D** *n* = 6–9). Expression of *Anp* and *Bnp* was determined in mouse hearts by qRT-PCR (**E**, *n* = 6–9). Scatter dot plot and mean, **p < 0.01, by two-way ANOVA and Tukey's *post-hoc* analysis for TUNEL and BAX quantification; mean and SEM, *p < 0.05, ***p < 0.001 by unpaired *t*-test for qRT-PCR; ctrl, control; wk, weeks.

Although a trend toward a transient decrease after 2 weeks of TH deprivation was noted, no significant alterations in the degree of fibrosis were identified by quantification of Sirius Red staining (Figures 4A,B) and visualization by two-photon and polarized light microscopy (Figures 4C,D). Moreover, apoptosis rate (Figures 5A,B) differed not between the three treatment groups after 2 weeks. However, after 4 weeks, TUNEL staining revealed an increase in apoptotic cell death and a significant increase in BAX protein expression was found in hearts of T4-treated TAC mice (Figures 5A–D), while fibrosis was not affected. Moreover, quantification of heart failure markers revealed an increase in cardiac atrial natriuretic peptide (*Anp*) and brain natriuretic peptide (*Bnp*) expression under T4 treatment compared to controls or TH-deprived animals (Figure 5E). Of note, hypothyroid hearts displayed the lowest *Anp* levels of all three treatments groups at the end of experiments, 5 weeks after TAC surgery (*p* < 0.001, Figure 5E). These changes in T4-treated TAC mice, however, were not accompanied by increased fibrosis, suggesting that T4 treatment might have diverged impact on cardiomyocyte apoptosis and cardiomyocyte function in heart.

### mTOR Signaling in Hearts Subjected to TAC Depends on TH Availability

**Figure 6:**
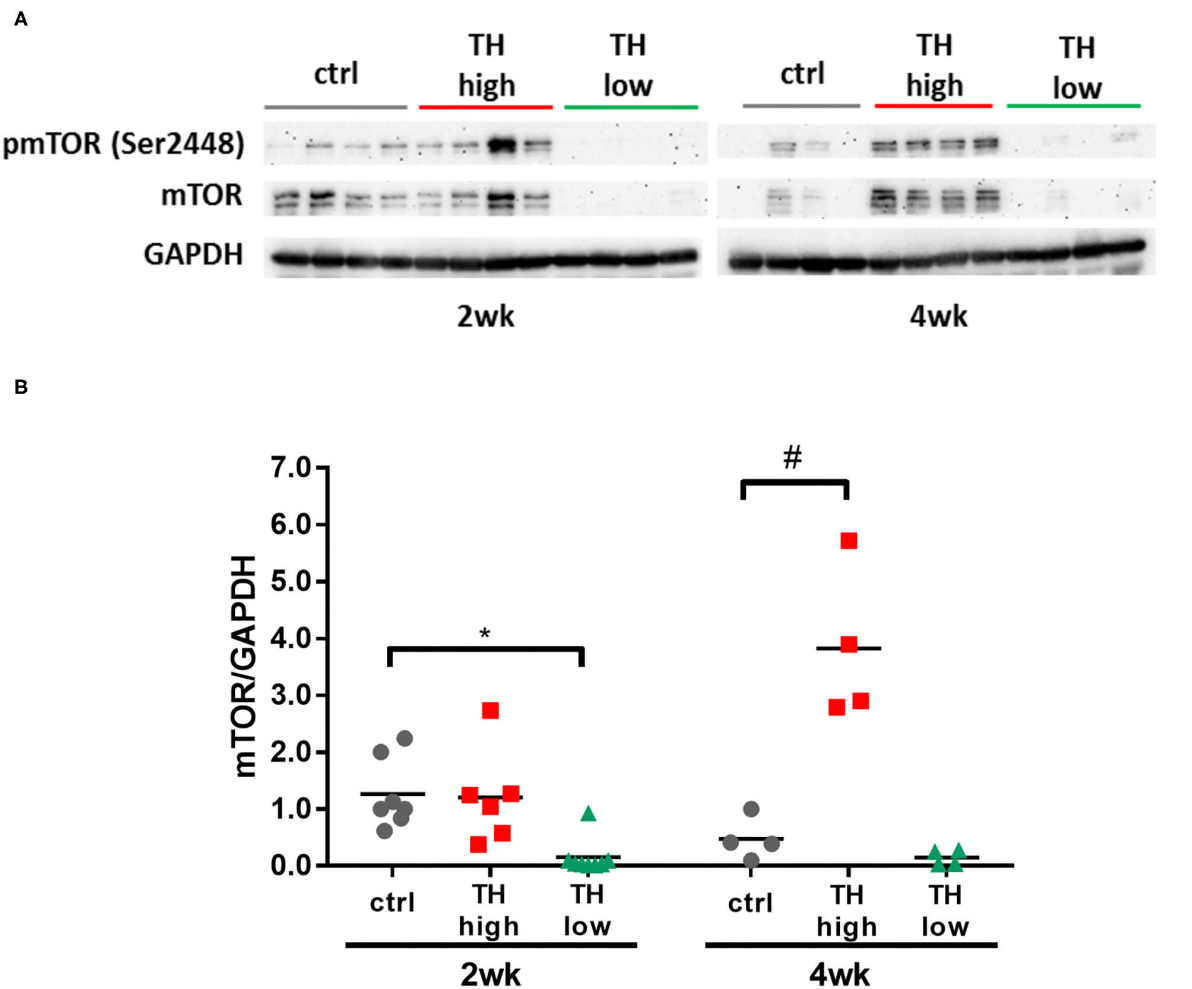
Cardiac expression of mTOR increased with T4 administration and decreased with TH deprivation. mTOR expression was significantly elevated after 4 weeks of T4 treatment, whereas TH deprivation resulted in drastic reduction of mTOR expression within 2 weeks (**A,B**, *n* = 4–8). Scatter dot plot and mean, *p < 0.05, ^#^*p* < 0.0001 by two-way ANOVA and Tukey's *post-hoc* analysis; ctrl, control; wk, weeks.

Several molecular pathways have been implicated in maladaptive cardiac hypertrophy (5, 35–37). One signaling pathway that is a key mediator of pathological growth and has recently gained increasing attention in the heart is the mTOR pathway (9, 38, 39). In fact, increased cardiac mTOR signaling has previously been demonstrated after TAC in mice (38, 40, 41). In our study, 4 weeks of T4 treatment resulted in significantly increased mTOR expression in TAC mouse hearts (Figure 6). Conversely, TH deprivation resulted in strongly reduced mTOR expression after 2 and 4 weeks in TAC mouse hearts (Figures 6A,B) compared to controls and T4-treated mice.

Other pathways that are important signaling transducers are the serine/threonine-specific protein kinase AKT and extracellular signal-regulated kinase 1/2 (ERK 1/2). Transient activation of both kinases, expressed by increased ratio of pAKT/AKT and pERK/ERK, was noted in hearts of TH-deprived mice (Supplementary Figure 3). However, 4 weeks of treatment of low or high TH condition did not activate these pathways.

### TH Availability Dominates the Regulation of Genes Involved in Cardiac Contractility

Cardiac contractility is determined by multiple factors in normal and failing hearts and several involved molecules are also described to be TH responsive (13, 42–44). To assess their regulation upon TAC-induced pressure overload in relation to TH serum status, transcript levels of cardiac contractility genes *Myh6, Myh7, Pln*, and *Serca2a* were quantified by qRT-PCR.

**Figure 7:**
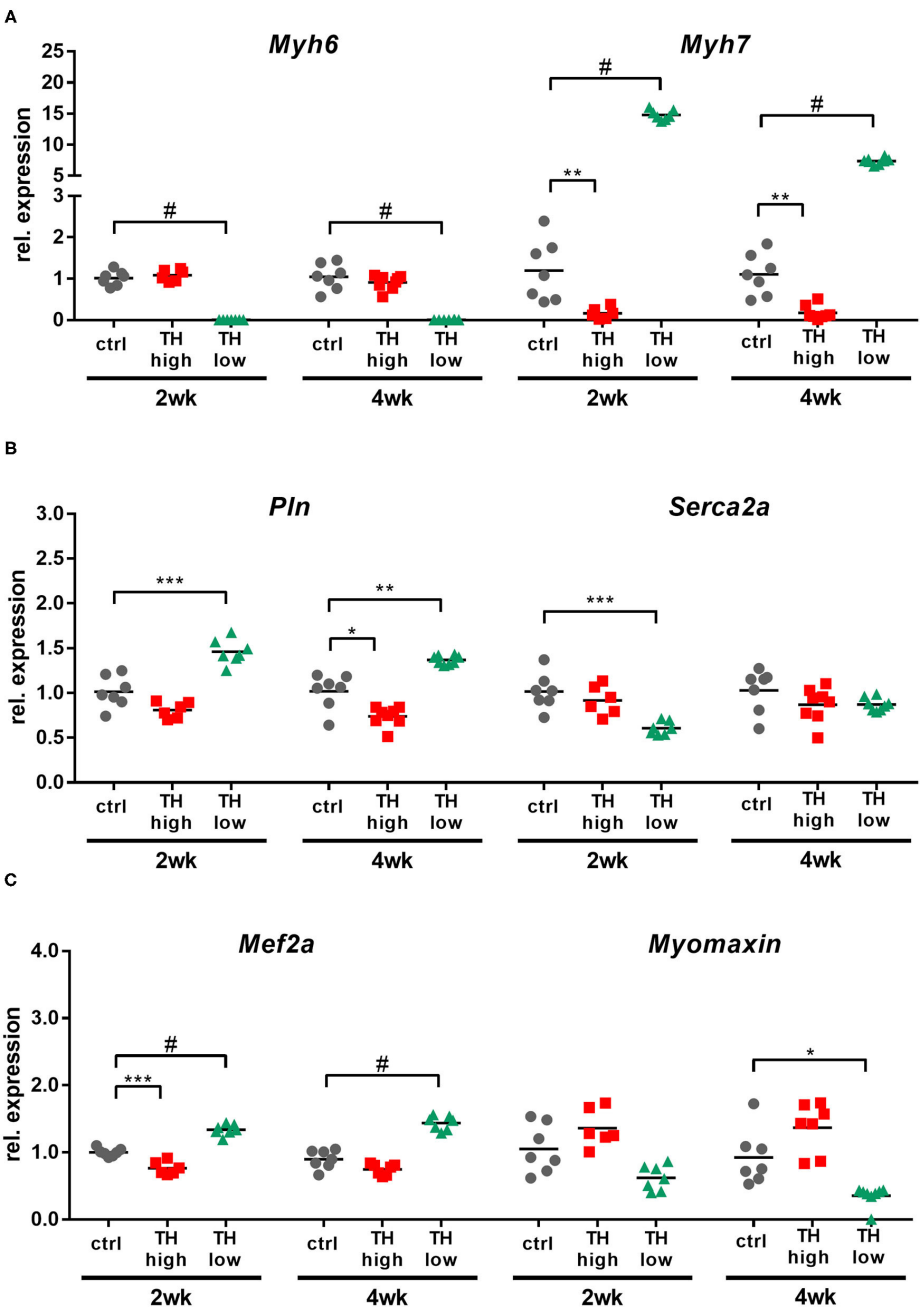
Cardiac expression of cardiac contractility genes and TH-regulated genes was primarily affected by high and low TH serum state. TH availability determined the transcript amounts of *Myh6, Myh7, Pln, Serca2a, Mef2a*, and *Myomaxin* in mouse hearts **(A–C)**. Scatter dot plot and mean, *p < 0.05, **p < 0.01, ***p < 0.001, and ^#^*p* < 0.0001 by unpaired *t*-test for qRT-PCR; ctrl, control; wk, weeks.

High TH availability repressed *Myh7* expression at 2 and 4 weeks and *Pln* expression after 4 weeks of treatment (Figures 7A,B), while TH deprivation increased transcript levels of *Myh7* and *Pln* and downregulated *Myh6* and *Serca2a*. In addition, expression of a gene regulating differentiation and calcium-dependent gene expression in muscle cells [*myocyte-specific enhancer factor* (*Mef2a*)] and its downstream target *Myomaxin* was investigated (Figure 7C). T4 treatment lowered *Mef2a* expression after 2 weeks, but not after 4 weeks. In contrast, low TH status elevated *Mef2a* transcripts at both time points. No significant changes were found for *Myomaxin* expression. Thus, TH status had a more pronounced impact on regulation of cardiac contractility genes than TAC-induced chronic pressure overload.

## Discussion

Chronic pressure overload of the left ventricle is a pathological condition and predictor of adverse cardiovascular events. It can be caused by systemic hypertension or aortic stenosis due to increased peripheral resistance or obstruction, respectively (45). Here, we have used the so-called “TAC model” to induce chronic left ventricular pressure overload in order to simulate a central aspect of hypertensive heart disease. Interestingly, in these conditions—different to expectations from some preclinical and clinical studies (5, 20, 46, 47)—we found that high TH treatment was not beneficial for diseased heart while TH deprivation was highly protective.

It was previously shown that hypercorrection of a hypothyroid-like state, which occurs in heart failure, e.g., by decreased TH receptor expression or increased deiodinase type 3 activity in heart, may restore cardiac function (20, 48–51). Similarly, systemic hypothyroidism may confer cardiovascular morbidity and mortality and hence is widely regarded as a TH substitution indication (52–54). However, recent studies challenge this “one-for-all” concept (55–57) and have advocated a more risk-based TH treatment strategy in case of hypothyroidism (58, 59). While most therapeutic goals are directed at reducing cardiovascular risk, treatment studies addressing changes in TH status and their impact on progression of disease in different cardiovascular disease entities are still missing (60, 61). In this study, in the presence of chronic pressure overload, 4 weeks of TH deprivation resulted in stop of pathological cardiac growth with decreased wall thicknesses and lowered heart weight-to-tibia length ratio and cardiomyocyte size compared to TAC controls. This was associated with an impressively improved cardiac function as illustrated by a 25% increased FS compared to controls and a 50% increased FS compared to T4 treated TAC mice. Furthermore, several parameters suggest a detrimental impact of TH treatment in our model of congestive heart failure. For example, lung weight-to-tibia length ratio as a measure of congestive heart failure increased after 2 weeks of T4 treatment, two death events occurred, and on the molecular level, this was reflected in increased cardiomyocyte apoptosis, mTOR activation, and increased expression of heart failure markers *Anp* and *Bnp* in hearts of T4-treated TAC mice. Thus, other than expected (20, 47, 50), T4 treatment did not prevent cardiac remodeling under chronic pressure overload but worsened the outcome.

One explanation for the discrepancy between the expected and observed impact of TH status on cardiac disease progression might be the heart failure model used in our study, besides the high T4 dosage, which was used to counteract the low TH heart state observed in subacute phase of mouse heart disease (62). Thus, vasodilation and subsequent reduction in peripheral resistance is one major cardiovascular effect of TH and improved cardiac output (63); however, in the TAC model, peripheral resistance is preset and stably induced by aortic ligation. Similarly, a vascular impact of hypothyroidism, which normally increases peripheral resistance (64), can be neglected in our study. In fact, in the clinical setting, there are patients who suffer from chronic left ventricular pressure overload, e.g., due to aortic stenosis or hypertrophic obstructive cardiomyopathy, similarly to the situation reflected in the TAC mouse model. In this condition, increased TH availability that mainly targets the heart within the cardiovascular system seems to induce cardiac cell death and deterioration of functional parameters.

Increased TH availability may also confer harmful effects on the heart by an increase in cardiac sympathetic stress (65–67). Thus, an altered adrenergic responsiveness at heart level could also contribute to the observed harmful effects of T4 treatment or conversely the cardioprotective effects of TH deficiency in hearts under chronic pressure overload.

While TH status-dependent changes in cardiac function in our TAC model were in line with increased or decreased cardiomyocyte apoptosis and up- or downregulation of mTOR signaling in the T4-treated and TH-deprived TAC mouse groups, respectively, future studies are needed to examine the role of mTOR signaling in the TH-dependent recovery from pressure overload using specific inhibitors.

TH-dependent cardiac hypertrophy was described as physiological hypertrophy mediated by the phosphoinositide 3-kinase/protein kinase B/mammalian target of rapamycin (PI3K/AKT/mTOR) signaling pathway (2, 9, 68). Over time, a compensated hypertrophy may result in left ventricular dilatation and develop pathological type of hypertrophy, which involves activation of additional intracellular signaling pathways such as ERK1/2, p38 MAPK, and JNK 1/2/3 (69) or the calcineurin system (70). Thus, previous studies described the activation of AKT and ERK upon TH treatment after myocardial infarction to be dose-dependent (71). Others found a TH-dependent ERK inhibition in pressure overload-induced mouse hearts (47). In our study, a transient activation of AKT and ERK signaling was noted after 2 weeks of TH deprivation and no change upon TH supply. However, due to the complex linkage and connection of intracellular signaling pathways during development and progression of cardiac hypertrophy, a dose- and time-dependent effect of TH is most likely.

Furthermore, no difference was found in the degree of fibrosis in the mouse hearts. The limited observation time of 4 weeks or anti-fibrotic effects of T4 reported by some groups (18, 72) may be reasons for the observation that the TH-dependent changes in TAC hearts did not (yet) translate into increased collagen deposition, a common histological finding in congestive heart failure.

Intrinsic effects on cardiac contractility such as changes in the expression of *Myh6, Myh7, Serca2a*, and *Pln* may determine function in normal and diseased hearts. Moreover, a switch involving decreased *Myh6* and *Serca2a* and increased *Myh7* and *Pln* transcripts was reported in conditions of hypertrophy and heart failure (42–44, 73, 74). Interestingly, this expression pattern was also found in the absence of TAC in hearts of hypothyroid mice while the opposite was observed in hyperthyroid mice in our previous experiments (13), suggesting that systemic TH availability may dominate the influence on cardiac gene expression rather than effects of pressure overload. In addition, TAC hearts under hyperthyroid conditions showed downregulation of TRα, while receptor expression was increased in TH-deprived *TAC* hearts, suggesting a local adaption of TH action in response to maladaptive hypertrophy and altered TH availability. In line, we found *Mef2a* to be similarly regulated as TRα most likely due to its function as a transcriptional co-factor of TRα (75). Decreased expression of *Mef2a* downstream-target Myomaxin was measured after 4 weeks in hypothyroid mice. This might be a protective state, as less myocardial damages when exposed to Angiotensin II were observed in mouse hearts with 20% of residual *Myomaxin* expression (76).

## Limitations

For our study, we have chosen relatively strong hyper- and hypothyroid states to evaluate the effects of dysregulated hormone action. Such dosages are usually used in other experimental mice studies (24, 29, 32) and are well described. However, more moderate doses or short-term derailment may have different impact on development and progression of pressure overload. In addition, hypothyroidism may have only a transient beneficial effect and could change into detrimental outcome in the long-term. Also, the impact of different TH conditions on signaling pathways under pressure overload need to be further investigated to better understand the underlying molecular events.

## Future Directions

Future animal studies should include more increments of TH states and treatment windows to fine-tune an optimal dosage as well as time frame. In the light of the outcome of our study, our data suggest that future clinical studies are warranted to define optimal thyroid status and reconsider differential TH therapy schemes in patients with cardiovascular disease conditions such as patients with aortic stenosis.

## Conclusion

In summary, we found that TH deprivation is beneficial for diseased heart under chronic pressure overload with regard to slowing down hypertrophic remodeling and improving cardiac contractile performance. This represents an early protective effect of hypothyroidism in the progression of maladaptive hypertrophy and prevents development of congestive heart failure. In contrast, our data underline that high-dose TH administration accelerates cardiac dysfunction under condition of maladaptive hypertrophy. The latter findings clearly underline the detrimental role of increased if not excessive TH availability in the diseased heart as has been stated in several recent guidelines on management of subclinical and overt thyroid dysfunction (56, 77) and illustrated in a recent heart failure population study (78).

## Data Availability Statement

The raw data supporting the conclusions of this article will be made available by the authors, without undue reservation.

## Ethics Statement

The animal study was reviewed and approved by LANUV AZ 84-02.04.2016.A261.

## Author Contributions

HK, ET, KL, and DF: material preparation, data collection, and analysis were performed. GH, JH-W, JG, and LM contributed to analysis and interpretation of data. The first draft of the manuscript was written by HK, KL, and DF. All authors commented on previous versions of the manuscript and contributed to the study conception, design, read, and approved the final manuscript.

## Conflict of Interest

The authors declare that the research was conducted in the absence of any commercial or financial relationships that could be construed as a potential conflict of interest.

## Publisher's Note

All claims expressed in this article are solely those of the authors and do not necessarily represent those of their affiliated organizations, or those of the publisher, the editors and the reviewers. Any product that may be evaluated in this article, or claim that may be made by its manufacturer, is not guaranteed or endorsed by the publisher.

## Abbreviations

ANP: atrial natriuretic peptide
BAX: Bcl2 associated protein X
BNP: brain natriuretic peptide
ECHO: echocardiography
FT3: free triiodothyronine
FT4: free thyroxine
FS: fractional shortening
GAPDH: glyceraldehyde-3-phosphate dehydrogenase
H&E: hematoxylin and eosin
IVSd: diastolic intraventricular septum thickness
LoI/MMI/${\text{ClO}}_{4}^{-}$: low iodine diet/methimazole/perchlorate
LVIDd: diastolic left ventricular inner diameter
LVPWd: diastolic left ventricular posterior wall thickness
mTOR: mechanistic target of rapamycin
MYH6: myosin heavy chain alpha
MYH7: myosin heavy chain beta
PLN: phospholamban
pmTOR: phosphorylated mechanistic target of rapamycin
T4: thyroxine
TAC: transverse aortic constriction
TH: thyroid hormones
TRα: thyroid hormone receptor alpha
TRβ: thyroid hormone receptor beta
TT4: total thyroxine
TUNEL: terminal deoxynucleotidyl transferase dUTP nick end labeling.
