# Supplementary material for: Protective Effects of Thyroid Hormone Deprivation on Progression of Maladaptive Cardiac Hypertrophy and Heart Failure
Source: Front Cardiovasc Med. 2021 Jul 30;8:683522. doi: 10.3389/fcvm.2021.683522 (PMC8363198; doi:10.3389/fcvm.2021.683522)
Supplement: Supplementary file 1 [file Table_1.docx]

Supplemental file

**Supplemental Table 1:** Oligonucleotides for quantitative RT-PCR. Oligonucleotides were designed using PrimerBlast (NCBI) and synthesized by Eurofins (Eurofins MWG Synthesis, Ebersberg, Germany).

| Gene name | Forward primer | Reverse primer |
| --- | --- | --- |
| *18S* | *CGGCTACCACATCCAAGGAA* | *GCTGGAATTACCGCGGCT* |
| *Polr2a* | *CTTTGAGGAAACGGTGGATGTC* | *TCCCTTCATCGGGTCACTCT* |
| *Gapdh* | *CCTCGTCCCGTAGACAAAATG* | *TGAAGGGGTCGTTGATGGC* |
| *Anp* | *TCGTCTTGGCCTTTTGGCTT* | *GGTGGTCTAGCAGGTTCTTGAAAT* |
| *Bnp* | *GTTTGGGCTGTAACGCACT* | *TCACTTCAAAGGTGGTCCCAG* |
| *Myh6* | *CAGACAGAGATTTCTCCAACCCA* | *GCCTCTAGGCGTTCCTTCTC* |
| *Myh7* | *CACGTTTGAGAATCCAAGGCTC* | *CTCCTTCTCAGACTTCCGCA* |
| *Pln* | *TTCATGCTCTGCACTGTGACG* | *GCCAAATGTGAGCTGTCTTCTTTT* |
| *Serca2a* | *AACTACCTGGAACAACCCGC* | *TCATGCAGAGGGCTGGTAGA* |
| *Mef2a* | *CTCCGAGCTTGCCTCCACAG* | *AAGCCTTGCAGGGCAGACAG* |
| *Myomaxin* | *AGGAATCTGCCTTTTTAAGTGACAA* | *ATTCGTCCGTGAAGTGATGC* |

**Supplemental Table 2:** Echocardiographic parameters of all time points. Values are represented as mean ± standard deviation, n=number of animals, nd=not determined. *p<0.05 compared to basal values, #p<0.05 compared to control group at the indicated time (3 or 5 weeks), by One-Way Anova and Tukey´s post hoc analysis.

| **8 weeks** | **basal (n=56)** | **1w TAC (n=47)** | **3w TAC control (n=16)** | **3w TAC TH high (n=16)** | **3w TAC TH low (n=17)** | **5w TAC control (n=8)** | **5w TAC TH high (n=9)** | **5w TAC TH low (n=9)** |
| --- | --- | --- | --- | --- | --- | --- | --- | --- |
| **Heart Rate [bpm]** | 577±66 | 588±83 | 581±72 | 576±63 | 536±39 | 602±89 | 559±85 | 518±27 |
| **IVSd [mm]** | 0.69±0.06 | 0.93±0.08* | 1.08±0.14* | 1.10±0.11* | 0.86±0.08*^,#^ | 1.02±0.09* | 1.04±0.05* | 0.79±0.05*^,#^ |
| **IVSs [mm]** | 1.07±0.10 | 1.27±0.09* | 1.40±0.12* | 1.42±0.15* | 1.23±0.12*^,#^ | 1.24±0.13* | 1.29±0.10* | 1.13±0.07 |
| **LVIDd [mm]** | 3.50±0.25 | 3.42±0.34 | 3.34±0.43 | 3.65±0.47 | 3.40±0.32 | 3.50±0.47 | 4.00±0.35* | 3.33±0.21 |
| **LVIDs [mm]** | 2.06±0.25 | 2.33±0.42* | 2.20±0.55 | 2.55±0.61* | 2.14±0.36 | 2.53±0.53* | 3.06±0.41* | 2.15±0.17 |
| **LVPWd [mm]** | 0.69±0.04 | 0.92±0.07* | 1.03±0.12* | 1.06±0.11* | 0.85±0.09*^,#^ | 0.97±0.07* | 1.03±0.04* | 0.78±0.04*^,#^ |
| **LVPWs [mm]** | 1.10±0.10 | 1.24±0.10* | 1.40±0.11* | 1.41±0.17* | 1.20±0.11*^,#^ | 1.27±0.09* | 1.35±0.11* | 1.11±0.09 |
| **EF [%]** | 72.83±5.67 | 61.38±8.83* | 64.51±11.68* | 58.80±12.33* | 68.24±7.57 | 55.66±9.03* | 47.49±7.56* | 65.96±4.54 |
| **FS [%]** | 41.25±4.81 | 32.44±6.09* | 34.95±8.11* | 31.11±8.24* | 37.51±5.76 | 28.54±5.57* | 23.64±4.52* | 35.46±3.51 |
| **LV Mass [mg]** | 77.28±9.45 | 113.10±19.63* | 131.93±28.91* | 156.07±30.36*^,#^ | 98.40±11.18*^,#^ | 129.69±26.15* | 167.23±17.94*^,#^ | 85.66±9.65^#^ |
| **LV Mass corrected [mg]** | 61.82±7.56 | 90.48±15.70* | 105.55±23.13* | 124.86±24.29*^,#^ | 78.72±8.94*^,#^ | 103.75±20.92* | 133.78±14.35*^,#^ | 68.53±7.72^#^ |
| **LV Vol d [µl]** | 51.29±8.63 | 48.89±12.04 | 46.55±15.85 | 57.78±17.11 | 48.04±10.67 | 52.45±18.06 | 70.76±15.24*^,#^ | 45.55±6.71 |
| **LV Vol s [µl]** | 14.09±4.38 | 19.73±9.08* | 18.04±12.84 | 25.74±14.13* | 15.83±6.33 | 24.65±13.30* | 37.89±12.91* | 15.50±3.24 |
| **AV Peak Velocity [mm/s]** | nd | -4533±378 | -4557±483 | -4626±368 | -4523±533 | -4190±656 | -4653±249 | -4459±393 |
| **AV Peak Pressure [mmHg]** | nd | 82.78±13.50 | 84.00±16.09 | 86.15±12.59 | 83.00±21.33 | 71.98±20.52 | 86.86±9.46 | 80.16±14.01 |

Supplemental figure legends:


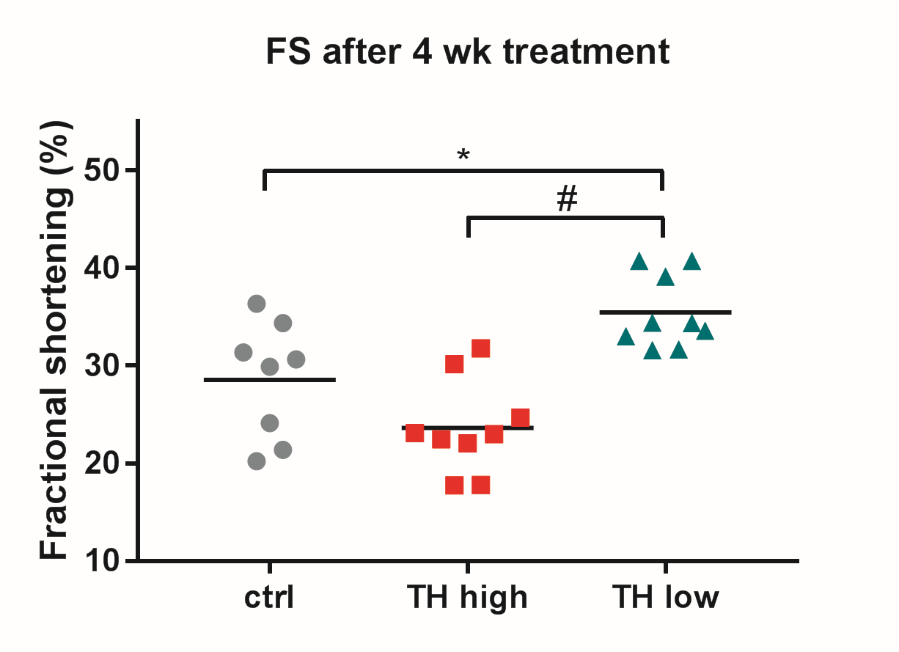


**Suppl. Fig. 1:** Fractional shortening after 4 weeks of treatment and in total 5 weeks after TAC surgery. TH low treated mice had an improved cardiac function at the end of experiment compared to control mice. *p<0.05, ^#^p<0.0001 determined by One-Way Anova.

**
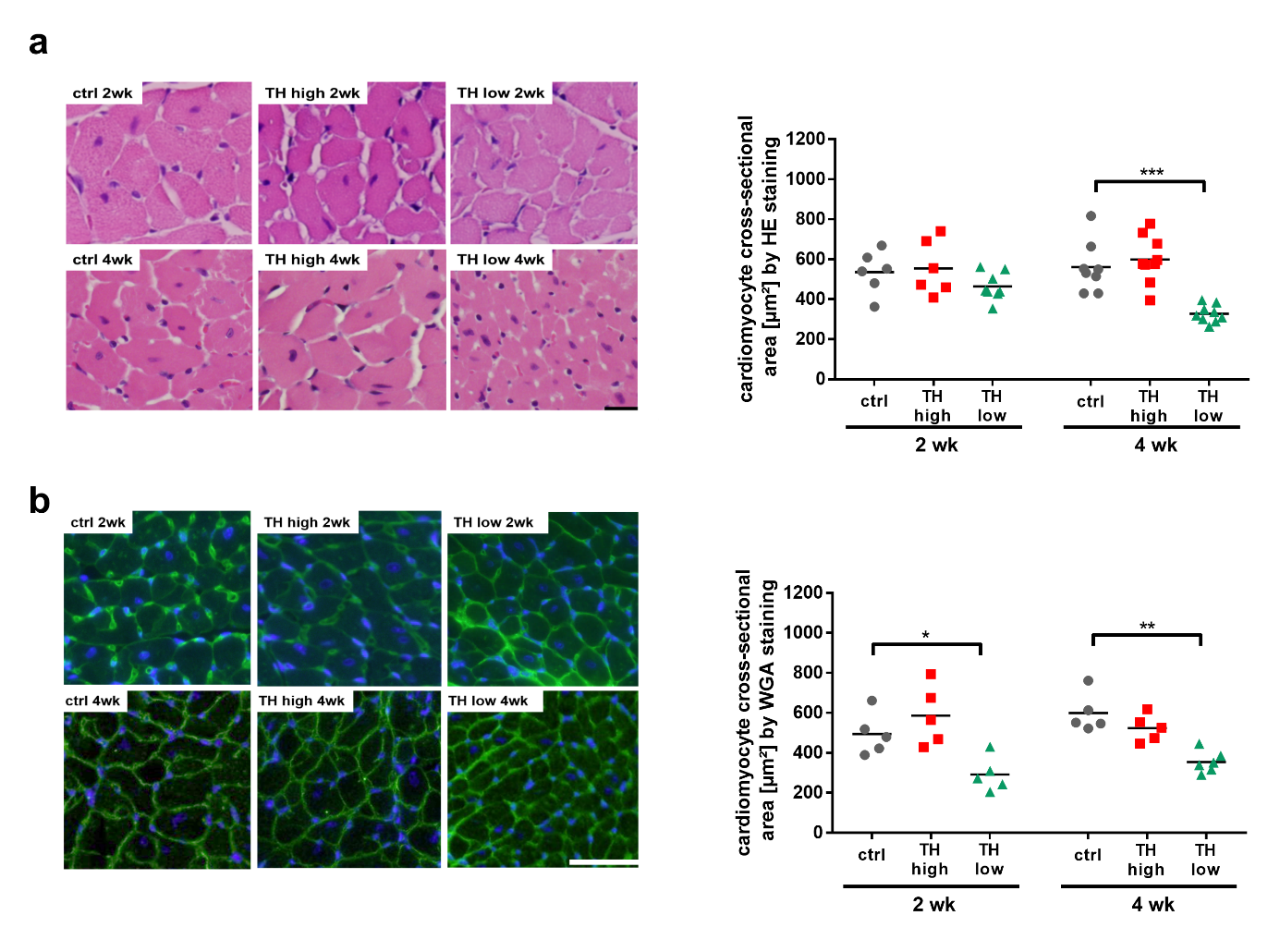
**

**Suppl. Fig. 2:** Representative pictures of H&E (a, scale bar=20 µm) and WGA (b, scale bar=50µm) stained sections of heart slices and quantification of cardiomyocyte cross-sectional area (n=5-9). TH deprivation significantly decreased cardiomyocyte size after two and four weeks of treatment. Scatter dot plot and mean, *p<0.05, **p<0.01, ***p<0.001 by Two-Way ANOVA and Tukey´s post hoc analysis; ctrl=control, wk=weeks.


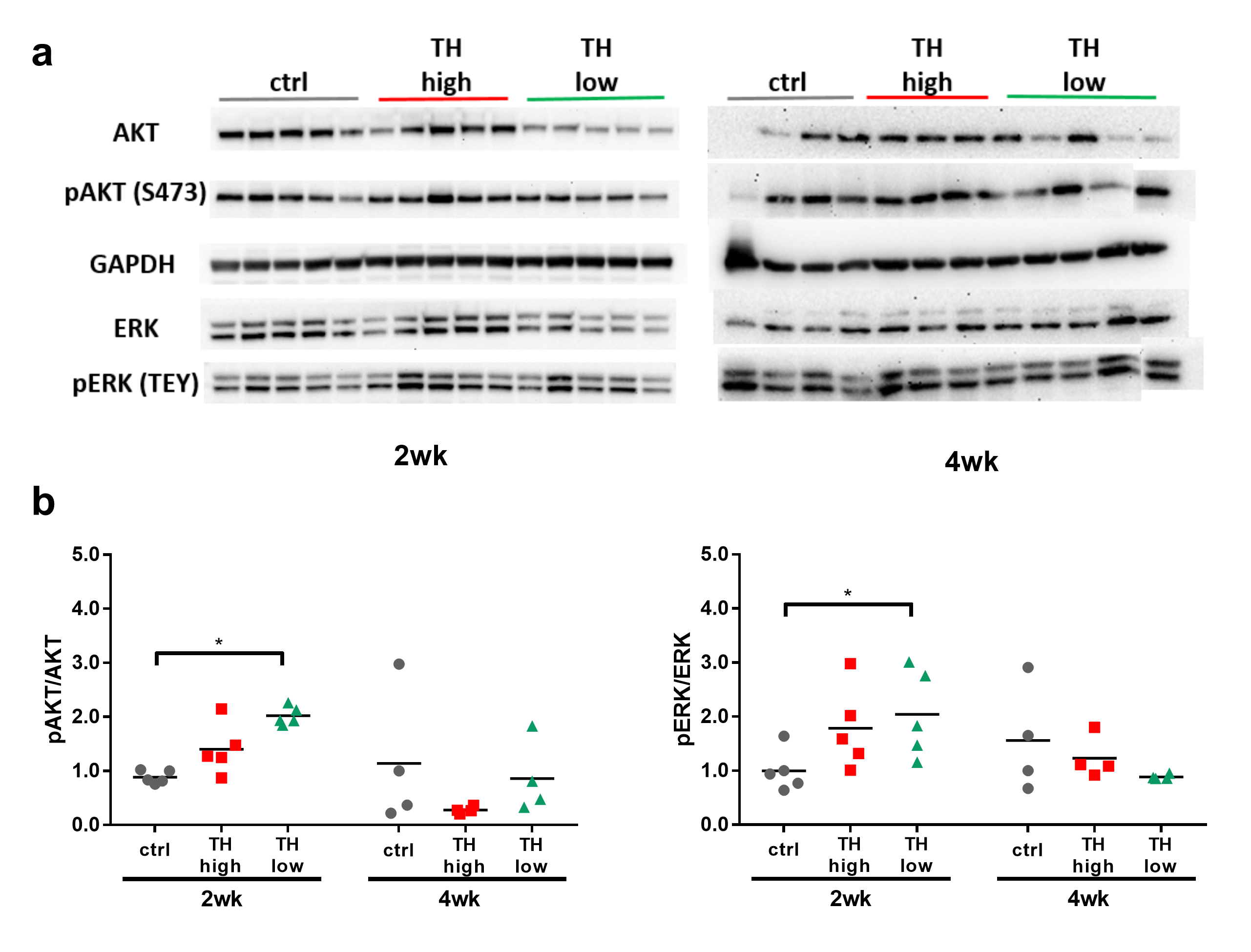


Suppl. Fig. 3: Cardiac protein expression of AKT and ERK kinases and respective phosphorylation sites. Upon low TH condition slightly elevated pAKT/AKT and pERK/ERK signals were noted. Scatter dot plot and mean, *p<0.05 by Two-Way ANOVA and Tukey´s post hoc analysis; ctrl=control, wk=weeks.
